# List of Reviewers 2025

**DOI:** 10.1017/jns.2026.10088

**Published:** 2026-04-01

The Editorial Board of *Journal of Nutritional Science* would like to thank the following for their contribution as peer reviewers in 2025:

Muiatu Abageda

Ajibola Ibraheem Abioye

Mahmood Ahmed

Fahmida Akter

Obed Akwaa Harrison

Sara Alsharif

Maryam Amini

Anchamo Anato

Lesley Andrade

Erik André

Mateus Dias Antunes

Seyyed Mostafa Arabi

Rosa Aragao Borner

Ulrike Arens-Azevedo

Rini Arianti

M. Arslan

Keiko Asakura

Nazlı Nur Aslan Çin

Erni Astutik

Fusta Azupogo

Paluku Bahwere

Ana Paula Bandeira de Oliveira

Katie Barfoot

Sanjay Basak

Natasha Bayes

Kristine Beaulieu

Mehrdad Behzadi

Goksel Bengi

Jonathan Bennett

Anteneh Berhane

Carissa van den Berk-Clark

Federicco Bernuzzi

Jessica Bihuniak

Anne Bjerregaard

Jorge Ble-Castillo

Makafui Borbi

Alejandro Borrego-Ruiz

Sharon Brownie

Iain Brownlee

Maria Buffini

Xuan Cai

Frederic Capel

Monica Carlsen

Lucrecia S. Carrera-Quintanar

Sherifath Chabi

Theresa Thompson Chaudhry

Gwen M. Chodur

Jeong-Hwa Choi

Divya Choudhary

Luis Colunga Lozano

Kristen Copeland

Matthias Corion

Laura Cornelsen

Marijana Curcic

Basma Damiri

Andrea Darling

Papa Dasari

Katie Davies

Carmen de la Rocha Martin del Campo

Giada De Palma

Vitor De Salles Painelli

Rosalie Delvert

Ramesh Dhakal

Aurup Ratan Dhar

Toluwalope Eyinla

Ngozika E. Ezinne

Amir Hossein Faghfouri

Diego Fano-Sizgorich

Khadijah Fayyaz

Fentaw Feleke

Léopold Fezeu

Mark Fox

Eduardo D. S. Freitas

Patrícia Freitas

Patience Gaa

Zsolt Gall

Tianlin Gao

Habtamu Gemede

Rachel Gibson

Mahdieh Golzarand

Keshav Gopal

Dmytro Gospodaryov

Antoneta Granic

Geoff Greene

Darren Greenwood

Xu-Guang Guo

Adyya Gupta

Dominika Guzek

Amir Hadi

Megan L. Hammersley

Slsayed Hamouda

Liisa Hantsoo

Natasha Haskey

Dan Hasson

Alyson Hill

Tom Hill

Sarah Hillier

Katy Horner

Ryota Hosomi

Yujiao Hua

Kazue Ishitsuka

Gaetano Isola

Priya Iyer

Laurie M. Jacobs

Rabia Javed

Isabela Thais Machado de Jesus

Angeline Jeyakumar

Haochen Ji

Peng Ji

Ming Jiang

Casey Johnson

Irmgard Jordan

Ralf Jäger

Toshihiro Kobayashi

Kanadice Kapinos

Avani Kapur

Ozgur Kasapcopur

John Kearney

Eda Keskin

Swati Kimothi

Frances Knight

Anika Knuppel

Akos Koller

Hamed Kord-Varkaneh

Junko Kose

Ingrida Kosiciarova

Sotiria Kotopoulou

Konsita Kuswara

Laura König

Wing-Fu Lai

Julie Lanigan

Svilena Lazarova

Lise Leblay

Catherine Leclercq

Jae-Gil Lee

Jia Yi Lee

Rebecca Leech

Heitor Leite

Jordan A. Levinson

Rebecca Lindberg

Shaojie Liu

Xin Liu

Jordan Llego

Dawid Madej

Hayato Maeda

Sergey A. Maksimov

Yuki Manabe

Alexandra Manson

Evangeline Mantzioris

Anthony Manyara

Chloe Marques

Stephanie Martin

Jorge Ivan Martinez

Andrew Matchado

Muniirah Mbabazi

Teresia Mbogori

Jeremy McCormick

Brandon McFadden

Aoibhin Moore Heslin

Alexandre Moreira

Nozomi Morikawa

Selekane Motadi

Megan Mueller

Daniel Mujuni

Angela Mulligan

Yuki Murakami

Ogechi Chinyere Nzeagwu

Mryam Nadery

Satish Natarajan

Elyas Nattagh-Eshtivan

Pablo A. Nava-Amante

Jeanne Neuffer-Frontout

Evie Nikokavoura

Hiroki Nishiwaki

Ilana Nogueira Bezerra

Shannon M. O’Connor

Cathal O’Hara

Sinead O’Mahony

Sreekanth Reddy Obireddy

Agartha Ohemeng

Sebastine Oiwoh

Akinkunmi Okekunle

Diriba Dereje Olana

Samuel A. Olowookere

Vahidreza Ostadmohammadi

Subramani Parasuraman

Manusha Paudel

Indira Paz Graniel

Lucia de Fátima Pedrosa

Nicoletta Pellegrini

Ying Peng

Hanno Pijl

Parul Puri

Michael Rak

Duarte Rener

Raj Rengalakshmi

Anouk Reuze

Cristian Ricci

Margaux Robert

Brenda Robles

Martina Rooney

Ida Rud

Yee-How Say

Lorena Saavedra-Garcia

Codjoe Samuel

Carlos Sepulveda

Mojtaba Shafiee

Hafiz Shahbaz

Mohammad Shannawaz

M. Kyla Shea

Akio Shimizu

Binam Raj Shrestha

M. Shahjalal Siam

Susan Sisson

Marek Skrzypski

Florian Slimano

Theodoros Smiliotopoulos

Jakub Sobiecki

Marie-Pierre St-Onge

Nelia Steyn

Maximilian Andreas Storz

Koki Sugimoto

Tiina Suikki

Ryoko Tajima

Xiaopeng Tang

Masresha Tessema

Grant Tinsley

Miguel Toribio-Mateas

Vanessa Trinca

Dereje Tsegaye

Ken Uechi

Tess van Rijssel

Katie Vasenina

Eliseu Verly Jr.

Stella Volpe

Thao-Uyen Vu

Qingchao Wang

Phillip Watson

Kyly Whitfield

Kathryn Whyte

Walter Willett

Katharina Wirnitzer

Keke Xiao

Ren-ying Xu

Akinori Yaegashi

Wai Yew Yang

Belete Yimer

Ruihong Yu

Xiaoyi Yuan

Ming Zhang

Xiang Zhang

Yumeng Zhang

Muhammad Zia

